# Hyperoside inhibits EHV-8 infection via alleviating oxidative stress and IFN production through activating JNK/Keap1/Nrf2/HO-1 signaling pathways

**DOI:** 10.1128/jvi.00159-24

**Published:** 2024-03-19

**Authors:** Tongtong Wang, Leyu Hu, Ruibo Li, Huiying Ren, Shuwen Li, Qi Sun, Xiangdan Ding, Yubao Li, Changfa Wang, Liangliang Li

**Affiliations:** 1 College of Agronomy, Liaocheng University, Liaocheng, China; 2 College of Veterinary Medicine, Qingdao Agricultural University, Qingdao, China; Lerner Research Institute, Cleveland Clinic, Cleveland, Ohio, USA

**Keywords:** hyperoside, EHV-8 inhibitor, JNK/Nrf2/ Keap1**/**HO-1, antioxidants, IFN response

## Abstract

Equine herpesvirus type 8 (EHV-8) causes abortion and respiratory disease in horses and donkeys, leading to serious economic losses in the global equine industry. Currently, there is no effective vaccine or drug against EHV-8 infection, underscoring the need for a novel antiviral drug to prevent EHV-8-induced latent infection and decrease the pathogenicity of this virus. The present study demonstrated that hyperoside can exert antiviral effects against EHV-8 infection in RK-13 (rabbit kidney cells), MDBK (Madin–Darby bovine kidney), and NBL-6 cells (E. Derm cells). Mechanistic investigations revealed that hyperoside induces heme oxygenase-1 expression by activating the c-Jun N-terminal kinase/nuclear factor erythroid-2-related factor 2/Kelch-like ECH-associated protein 1 axis, alleviating oxidative stress and triggering a downstream antiviral interferon response. Accordingly, hyperoside inhibits EHV-8 infection. Meanwhile, hyperoside can also mitigate EHV-8-induced injury in the lungs of infected mice. These results indicate that hyperoside may serve as a novel antiviral agent against EHV-8 infection.

**IMPORTANCE:**

Hyperoside has been reported to suppress viral infections, including herpesvirus, hepatitis B virus, infectious bronchitis virus, and severe acute respiratory syndrome coronavirus 2 infection. However, its mechanism of action against equine herpesvirus type 8 (EHV-8) is currently unknown. Here, we demonstrated that hyperoside significantly inhibits EHV-8 adsorption and internalization in susceptible cells. This process induces HO-1 expression via c-Jun N-terminal kinase/nuclear factor erythroid-2-related factor 2/Kelch-like ECH-associated protein 1 axis activation, alleviating oxidative stress and triggering an antiviral interferon response. These findings indicate that hyperoside could be very effective as a drug against EHV-8.

## INTRODUCTION

Equine herpesvirus type 8 (EHV-8) is one of the most important viral pathogens affecting horses and donkeys and can cause abortion, severe respiratory disease, and viral encephalitis (1, 2). Every year, EHV-8 infections contribute to tremendous economic losses in the equine industry. EHV-8, a double-stranded enveloped DNA virus with a 150 kb genome, belongs to the *α-herpesvirinae* subfamily (3). The 2019 outbreak of EHV-8 in some donkey farms in China triggered a wave of spontaneous abortions in pregnant donkeys, seriously retarding the growth of the donkey industry (2, 4). However, at present, there are few effective vaccines or drugs for the control of EHV-8. Therefore, more effective drugs against EHV-8 need to be developed.

Oxidative stress injury and type I interferon (IFN) responses are common in virus-infected cells. Heme oxygenase-1 (HO-1) is an effective cytoprotective enzyme with antioxidant and anti-inflammatory properties and can regulate type I IFN production during viral infections (5). Lu et al. found that piperlongumine inhibits Zika virus proliferation through HO-1-mediated oxidative stress relief (6). In addition, Feng et al. demonstrated that porcine reproductive and respiratory syndrome virus (PRRSV) suppresses type I IFN responses by negatively regulating HO-1 transcription via HOXA3 induction (7). However, the relationship between EHV-8 infection and HO-1 expression is currently unclear.

Hyperoside, an active compound isolated from *Rhododendron brachycarpum* G.Don (Ericaceae) (8), is believed to have antioxidant, antiviral, anti-inflammatory, antihyperglycemic, and anticoagulant properties (9–11). Park et al. showed that hyperoside protects against oxidative stress in human lens epithelial cells via HO-1 induction. This effect is mediated by the activation of extracellular signal-regulated kinase (ERK)/nuclear factor erythroid-2-related factor 2 (Nrf2) signaling (12). Hyperoside has also been reported to suppress herpesvirus (HSV), hepatitis B virus (HBV), infectious bronchitis virus, and severe acute respiratory syndrome coronavirus 2 (SARS-CoV-2) infection (13–17). However, the antiviral activity and potential molecular effects of hyperoside against EHV-8 are still unknown.

In the present study, the anti-EHV-8 activity of hyperoside was investigated *in vitro* and *in vivo*. Furthermore, the potential mechanisms underlying the anti-EHV-8 effects of hyperoside were uncovered. Our results revealed that hyperoside can effectively exert resistance against EHV-8 activity, both in susceptible cells and in mouse models. Further analysis demonstrated that hyperoside can increase HO-1 expression via the c-Jun N-terminal kinase (JNK)/Nrf2/Kelch-like ECH-associated protein 1 (Keap1) axis, eliciting antiviral IFN responses and alleviating oxidative stress to suppress EHV-8 replication. Our data indicate that hyperoside holds promise as a novel therapeutic agent against EHV-8.

## RESULTS

### Hyperoside does not exert cytotoxicity *in vitro*

**Fig 1:**
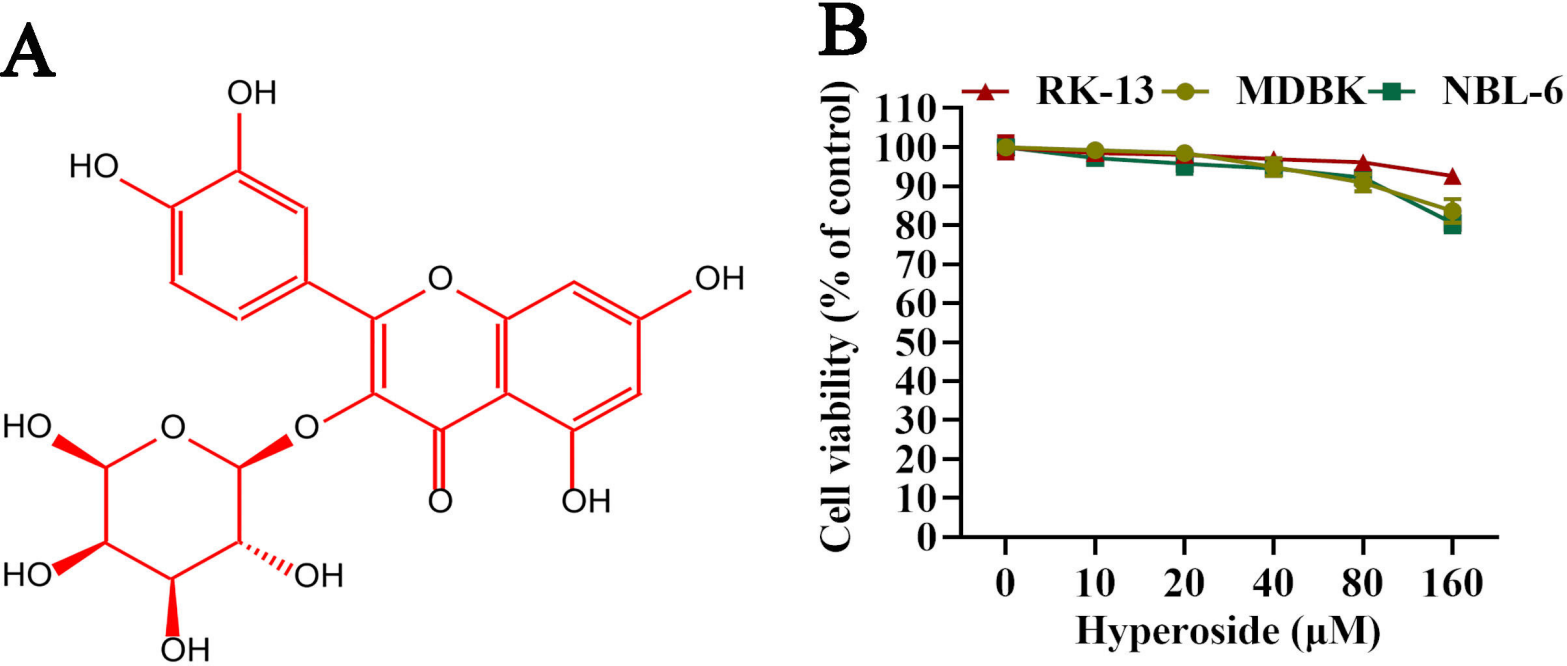
Chemical structure and cytotoxicity of hyperoside. (**A**) The chemical structure of hyperoside. (**B**) The cytotoxicity of hyperoside in RK-13, MDBK, and NBL-6 cells. Cells were preseeded in 96-well plates and treated with hyperoside (0, 10, 20, 40, 80, and 160 µM) for 24 h, and cell viability was measured using a CCK-8 assay. The data shown are representative of three independent experiments.

The structure of hyperoside is illustrated in Fig. 1A. In this study, we used the cell counting kit-8 (CCK-8) assay to test the potential cytotoxicity caused by various concentrations of hyperoside in RK-13, NBL-6, and MDBK cells. The maximal safe concentration of hyperoside in these cells was 80 µM. The relative viability of RK-13, NBL-6, and MDBK cells showed no significant change after treatment with 80 µM hyperoside (Fig. 1B).

### Hyperoside inhibits EHV-8 infection in susceptible cells

**Fig 2:**
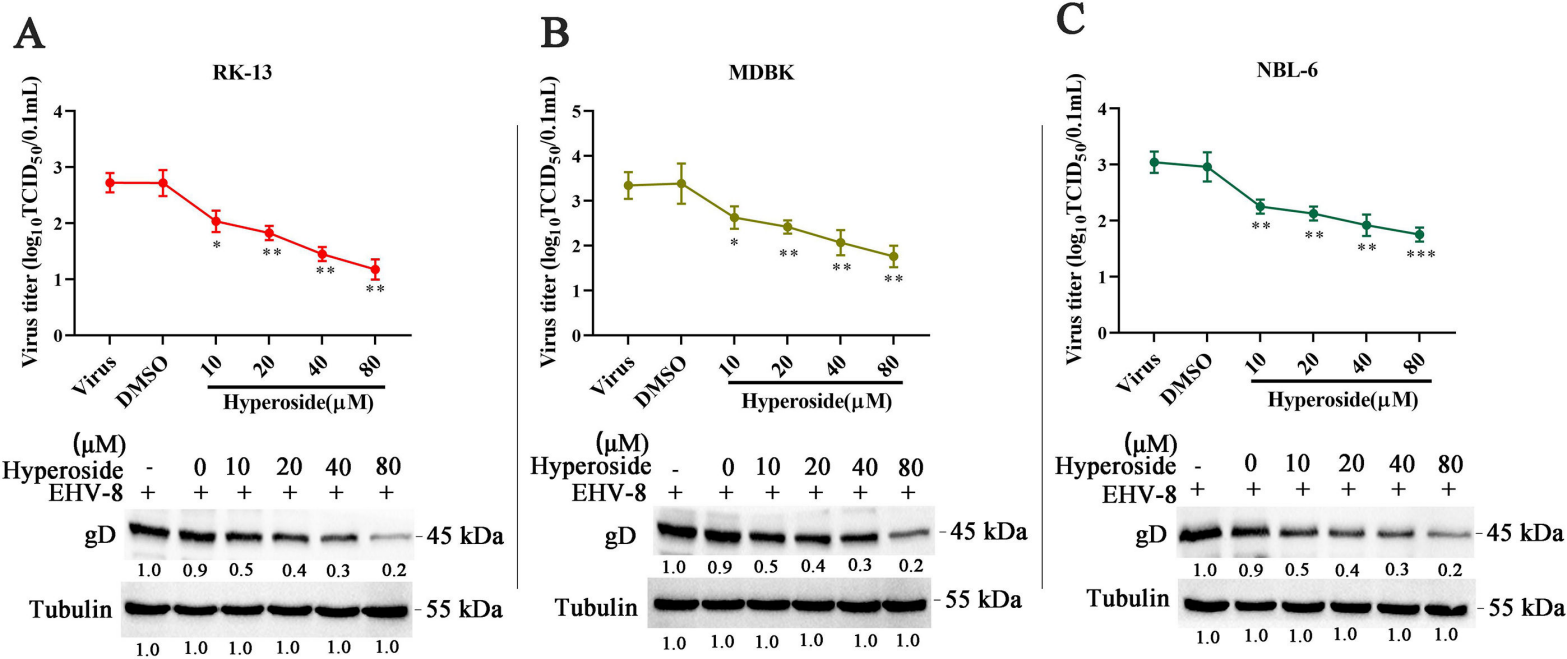
Antiviral activity of hyperoside against EHV-8 in susceptible cells. (**A**) RK-13 cells were infected with EHV-8 SDLC66 [0.1 multiplicity of infection (MOI)] after preincubation with hyperoside (10, 20, 40, and 80 µM) or dimethylsulfoxide (DMSO) (represented as 0 µM), respectively. The production of EHV-8 progeny and levels of the EHV-8 gD protein were measured using TCID_50_ and Western blot at 36 hpi. Furthermore, the antiviral effect of hyperoside was also confirmed in MDBK cells (**B**) and NBL-6 cells (**C**) using the same protocol. The data shown are representative of three independent experiments and were analyzed using one-way ANOVA. ^***^P < 0.001, compared with 0 µM hyperoside-treated cells.

**Fig 3:**
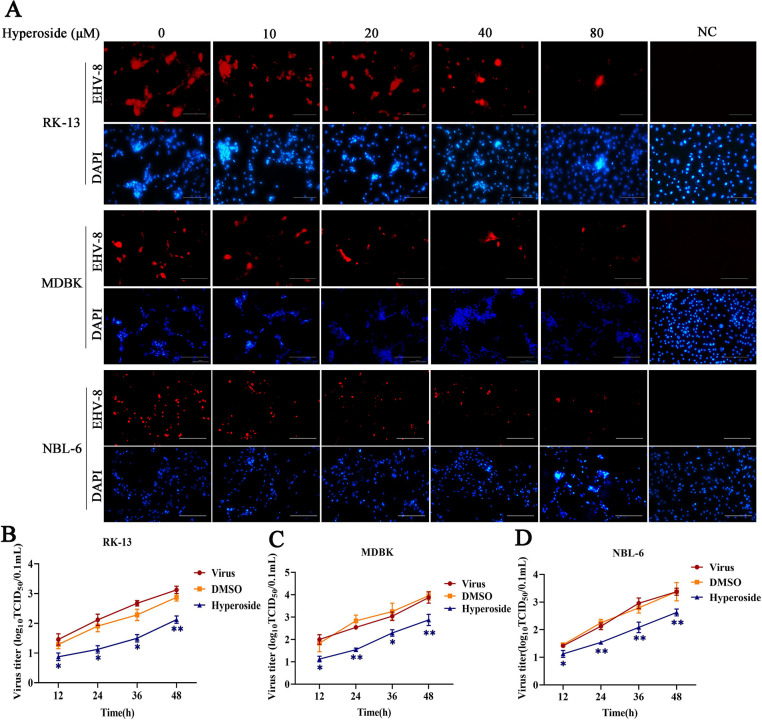
Hyperoside inhibited EHV-8 infection in susceptible cells. (**A**) RK-13, MDBK, and NBL-6 cells were pretreated with the indicated concentrations of hyperoside or DMSO for 2 h at 37°C. This was followed by incubation with EHV-8 SDLC66 at 0.1 MOI in the presence of hyperoside (0, 10, 20, 40, or 80 µM). These cells were fixed and stained with anti-EHV-8 serum to detect EHV-8 particles at 36 hpi (red). The nuclei were counterstained with 4′-6-diamidino-2-phenylindole (DAPI) (blue). Images were captured using a Leica microsystems fluorescence microscope (DMi8, Germany). Scale bar, 50 µM. In addition, the susceptible cells were infected with EHV-8 SDLC66 (0.1 MOI) in the presence or absence of hyperoside (80 µM), and cellular supernatants were collected at 12, 24, 36, and 48 hpi. The titer of EHV-8 progeny in RK-13 (**B**), MDBK (**C**), and NBL-6 (**D**) cells was calculated using the Reed–Muench method. ^*^P < 0.05; ^**^P < 0.01, compared with DMSO-treated cells.

To evaluate the antiviral activity of hyperoside, RK-13, MDBK, and NBL-6 cells were treated with different concentrations of hyperoside. Furthermore, the 50% tissue culture infectious dose (TCID_50_) and Western blot assays were performed to analyze virus replication at 36 hpi. Hyperoside could suppress EHV-8 gD protein expression and viral progeny production in RK-13, MDBK, and NBL-6 cells in a dose-dependent manner (Fig. 2A through C). Similar results were observed in the immunofluorescence assay (IFA) with anti-EHV-8 mouse serum (Fig. 3A).

To test the time course of hyperoside-induced antiviral effects, the RK-13, MDBK, and NBL-6 cells were pretreated with hyperoside (80 µM) for 2 h and then infected with EHV-8 SDLC66 (MOI = 0.1). The culture supernatants were examined to measure viral titers at the indicated time points. Compared to mock treatment, hyperoside treatment produced a significant reduction in viral titers at 12, 24, 36, and 48 hpi in RK-13 (Fig. 3B), MDBK (Fig. 3C), and NBL-6 (Fig. 3D) cells. These data indicated that hyperoside potently suppresses EHV-8 infection in susceptible cells *in vitro*.

### Hyperoside inhibits infection with other EHV-8 strains

**Fig 4:**
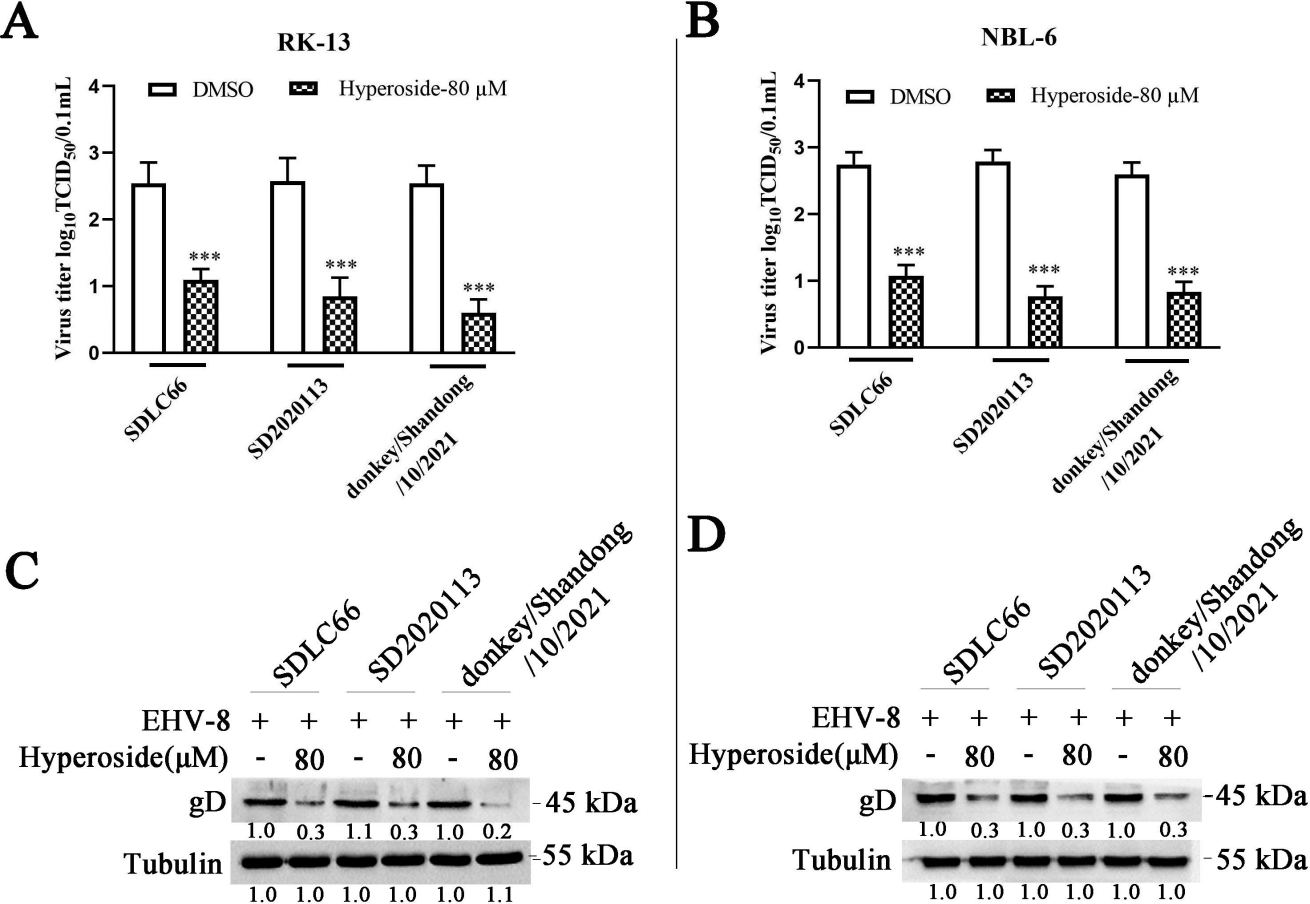
Hyperoside inhibited infection with other strains of EHV-8 in susceptible cells. RK-13 and NBL-6 cells were incubated with or without hyperoside (80 µM) for 2 h and then infected with the SDLC66, SD2020113, or donkey/Shandong/10/2021 strains of EHV-8 for 1 h at 37°C. The EHV-8 replication at 24 hpi was evaluated based on the TCID_50_ value in RK-13 cells (**A**) and NBL-6 cells (**B**). ^***^P  <  0.001 compared with DMSO-treated cells challenged with the same virus. Meanwhile, RK-13 and NBL-6 cells were also treated with hyperoside and different EHV-8 strains using the same protocol. The gD protein expression was tested using Western blot in RK-13 cells (**C**) and NBL-6 cells (**D**).

We further tested the antiviral activity of hyperoside (80 µM) against other EHV-8 strains—such as SD2020113 and donkey/Shandong/10/2021—in RK-13 and NBL-6 cells. TCID_50_ and Western blot assays were employed to evaluate EHV-8 replication. EHV-8 progeny production was significantly lower in RK-13 cells treated with hyperoside than in those treated with DMSO (Fig. 4A). Similar results were observed in NBL-6 cells (Fig. 4B). Consistent with the results of the TCID_50_ assay, gD protein expression was significantly downregulated in hyperoside-treated cells (Fig. 4C and D). Taken together, the findings showed that hyperoside possesses broad-spectrum anti-EHV-8 infection activity *in vitro*.

### Hyperoside shows antiviral activity in the initial stage of EHV-8 infection

**Fig 5:**
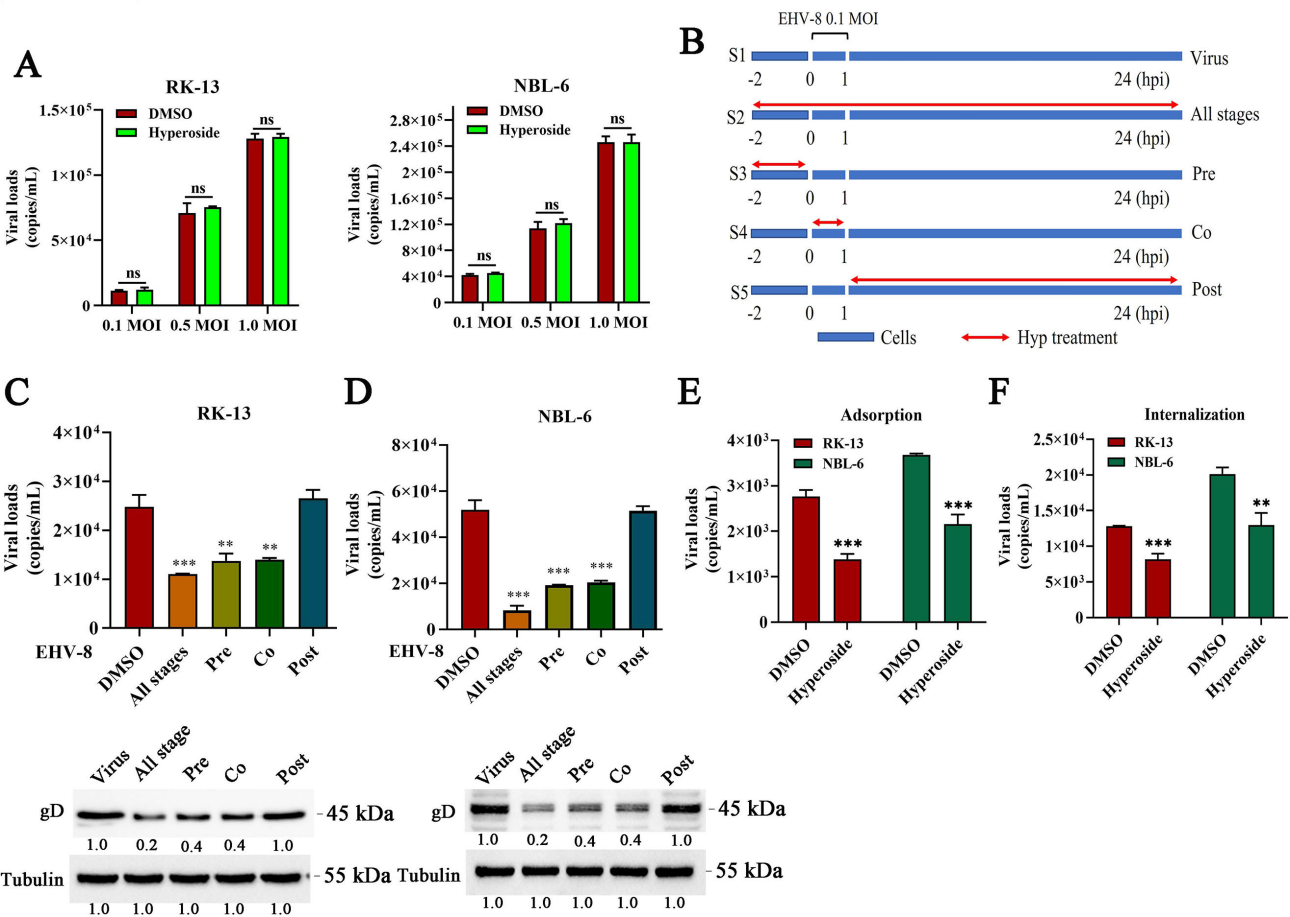
Hyperoside inhibited EHV-8 infection at the initial viral entry stage. (**A**) EHV-8 particles (0.1, 0.5, and 1 MOI) were incubated with hyperoside (80 µM) or DMSO for 2 h at 37°C. The virus was then inoculated into RK-13 and NBL-6 cells, and viral progeny was detected at 24 hpi using quantitative polymerase chain reaction (qPCR) to evaluate the virucidal activity of hyperoside. ns, not significant. (**B**) Time-of-addition schematic. The RK-13 and NBL-6 cells were treated with hyperoside (80 µM) at different time points relative to EHV-8 SDLC66 (0.1 MOI) infection, including pretreatment, co-treatment, post-treatment, and treatment at all stages. The cellular supernatants and cells were collected at 24 hpi, and viral replication in RK-13 (**C**) and NBL-6 (**D**) cells was further analyzed using qPCR and Western blot. ^**^P < 0.01; ^***^P  <  0.001, compared with DMSO-treated cells. (**E**) Adsorption assay. RK-13 and NBL-6 cells were incubated with a mixture of hyperoside or DMSO and EHV-8 (0.1 MOI) for 1 h at 4°C, and the cells were then washed. After incubation at 37°C for 24 h, the copy number of progeny virus particles was tested using qPCR. ^***^P < 0.001, compared with DMSO-treated cells. (**F**) Internalization assay. RK-13 and NBL-6 cells were pretreated with hyperoside (80 µM) for 12 h and then incubated with EHV-8 (0.1 MOI) at 4°C for 1 h. The cells were washed and finally incubated with 80 µM hyperoside or DMSO for another 1 h at 37°C. The copy number of progeny virus particles was detected using qPCR at 24 hpi. ^**^P < 0.01; ^***^P  <  0.001, compared with DMSO-treated cells.

A direct inactivation assay was performed to test whether hyperoside could directly inactivate EHV-8 in RK-13 and NBL-6 cells. The number of progeny viral particles in the hyperoside-treated EHV-8 infection group was not significantly different from that in the DMSO-treated EHV-8 infection group (Fig. 5A). This indicated that hyperoside has no direct virucidal effect on EHV-8. Subsequently, a time-of-addition experiment was conducted to determine which stage of the EHV-8 life cycle is affected by hyperoside (Fig. 5B). For this experiment, the cells were divided into various groups, as follows: S1 group, EHV-8 infection only (positive control); S2 group, hyperoside treatment at all stages, including pretreatment, co-treatment, and post-treatment; S3 group, hyperoside pretreatment only (Pre); S4 group, hyperoside and EHV-8 co-treatment (Co); and S5, hyperoside post-treatment only (Post). The cells were harvested at 24 hpi, and the mRNA and protein expression of gD was measured. As shown in Fig. 5C and D, gD expression was significantly lower in the S3 and S4 groups than in the S1 control group, indicating that hyperoside inhibits the early stages of EHV-8 infection. Subsequently, we performed viral adhesion experiments and virus entry assays to determine whether the antiviral activity of hyperoside is related to the suppression of viral adsorption or internalization in RK-13 and NBL-6 cells. These data showed that treatment with hyperoside resulted in a decrease in EHV-8 copy number in RK-13 and NBL-6 cells (versus the DMSO-treated control) (Fig. 5E). Similar results were also observed in RK-13 and NBL-6 cells during the internalization stage (Fig. 5F). These findings indicated that hyperoside inhibits EHV-8 infection at the adsorption and internalization stages.

### Hyperoside requires HO-1 to suppress EHV-8 replication

**Fig 6:**
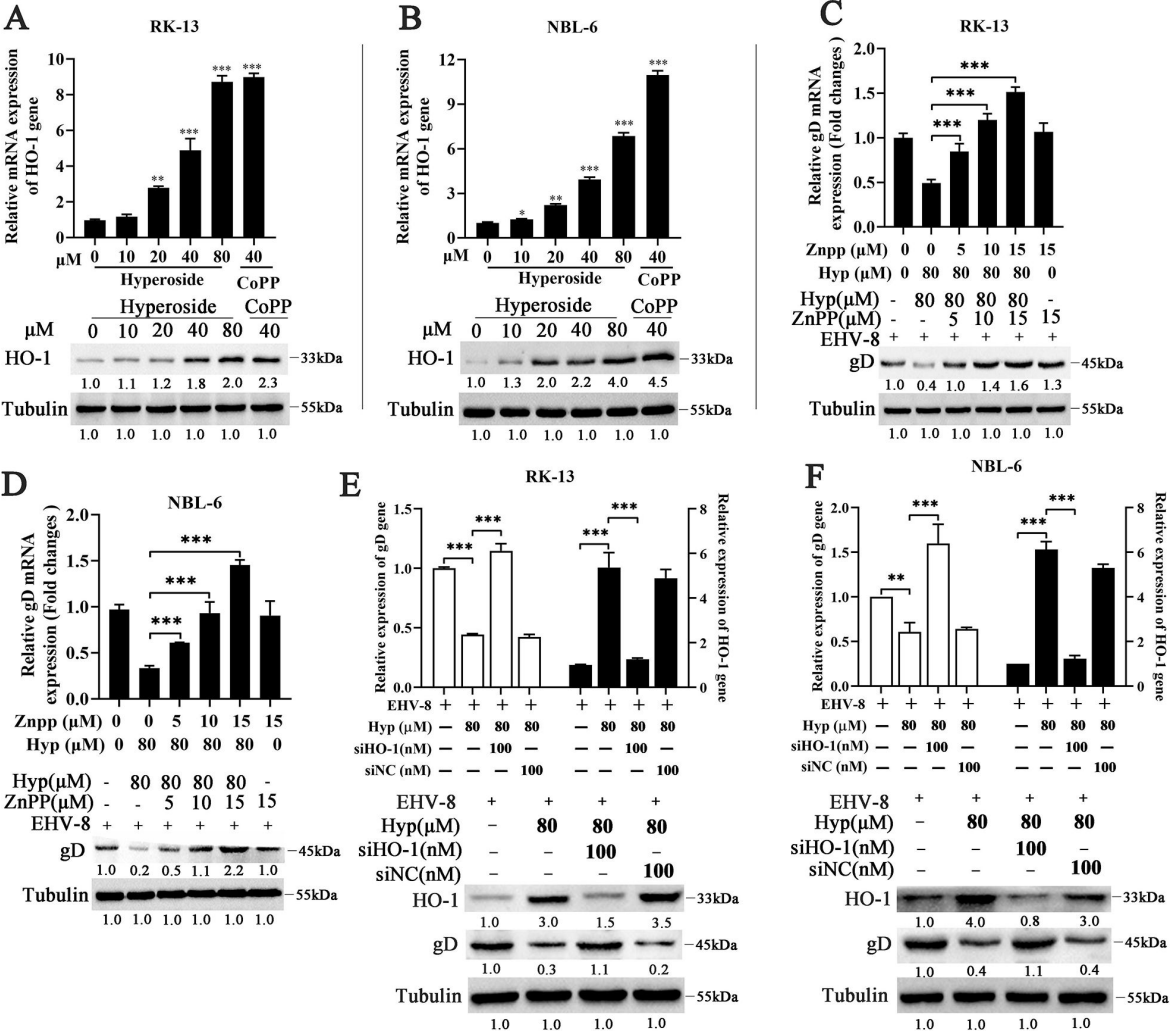
Hyperoside upregulated HO-1 expression to suppress EHV-8 replication. RK-13 cells were treated with various concentrations of hyperoside or cobalt protoporphyrin (CoPP) (40 µM) for 24 h and then harvested to extract RNA and protein. HO-1 expression was further analyzed with qPCR and Western blot. (**A**) ^**^P < 0.01; ^***^P < 0.001, compared with 0 µM hyperoside-treated cells. NBL-6 cells were also treated with hyperoside using the same protocol. HO-1 expression in these cells was determined using qPCR and Western blot (**B**). ^*^P < 0.05; ^**^P < 0.01; ^***^P < 0.001, compared with 0 µM hyperoside-treated cells. RK-13 and NBL-6 cells were co-treated with hyperoside (80 µM) and various concentrations of zinc protoporphyrin (ZnPP) for 2 h. They were then infected with EHV-8 SDLC66 (0.1 MOI) for 24 h, and the HO-1 and gD expression levels in RK-13 (**C**) and NBL-6 (**D**) cells were evaluated using qPCR and Western blot. ^***^P < 0.001, compared with 80 µM hyperoside-treated cells without ZnPP. Meanwhile, RK-13 (**E**) and NBL-6 (**F**) cells were transfected with siHO-1 or siNC for 12 h and then infected with EHV-8 SDLC66 (0.1 MOI) in the absence or presence of hyperoside (80 µM) for 24 h. HO-1 and gD expression levels were tested using qPCR and Western blot. glyceraldehyde-3-phosphate dehydrogenase (GAPDH) was used as the control. ^*^P < 0.05; ^**^P < 0.01; ^***^P < 0.001, compared with 80 µM hyperoside-treated cells without siHO-1 and siNC.

Hyperoside has previously been reported to exert antioxidant, anti-inflammatory, and antiviral effects (13, 18, 19). HO-1 is essential for cytoprotection against oxidative injury and other cellular stresses in various mammalian cells (20, 21). To determine whether hyperoside can modulate HO-1 expression, RK-13 and NBL-6 cells were treated with various concentrations of hyperoside for 24 h, and the HO-1 expression levels in these cells were examined using qPCR and Western blot. As expected, both the mRNA and protein levels of HO-1 increased significantly after hyperoside treatment in RK-13 cells (Fig. 6A) and NBL-6 cells (Fig. 6B).

To test whether the anti-EHV-8 activity of hyperoside is related to HO-1 activation, RK-13 and NBL-6 cells were co-treated with hyperoside (80 µM) and different concentrations of ZnPP. The cells were then infected with EHV-8 SDLC66. EHV-8 replication was detected using qPCR and Western blot at 24 hpi. The results showed that hyperoside significantly increased HO-1 expression and reduced gD expression. Meanwhile, ZnPP attenuated the anti-EHV-8 activity of hyperoside in RK-13 (Fig. 6C) and NBL-6 (Fig. 6D) cells.

In another series of experiments, RK-13 and NBL-6 cells were transfected with an siRNA targeting HO-1 (siHO-1), treated with hyperoside (80 µM), and infected with EHV-8 SDLC66 (MOI = 0.1). Subsequently, HO-1 and gD expression was analyzed using qPCR and Western blot. siHO-1 was found to reverse the anti-EHV-8 effect of hyperoside in both RK-13 (Fig. 6E) and NBL-6 (Fig. 6F) cells. Collectively, these findings suggested that the anti-EHV-8 activity of hyperoside depends on HO-1 activation.

### Hyperoside alleviates EHV-8-induced oxidative stress via HO-1 activation

**Fig 7:**
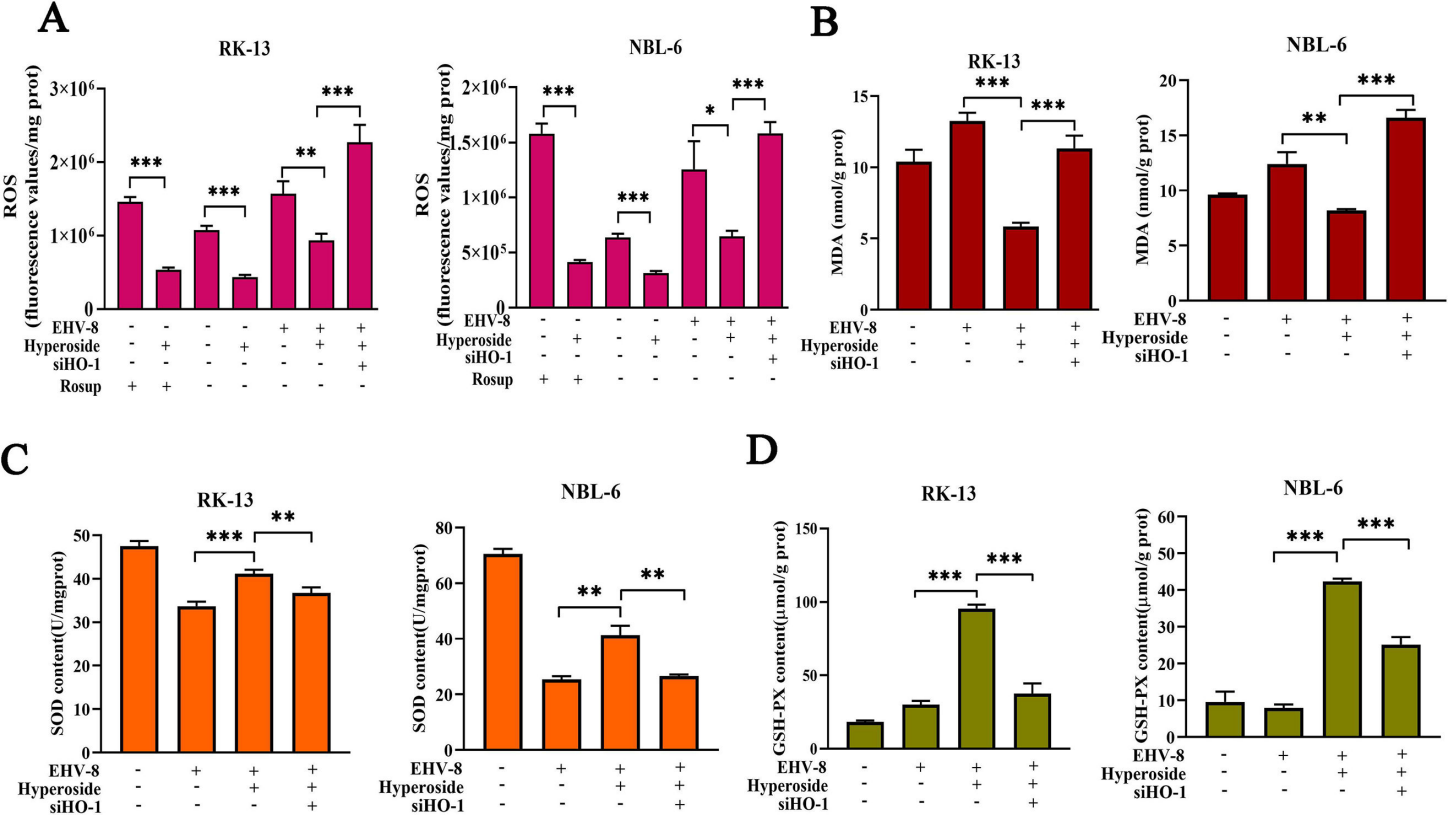
Hyperoside attenuated the oxidative stress induced by EHV-8 via HO-1 regulation. RK-13 and NBL-6 were transfected with siHO-1 for 10 h, pretreated with 80 µM hyperoside for 2 h, and then infected with EHV-8 (0.1 MOI) for another 1 h. ROS generation in RK-13 and NBL-6 cells was determined using the DCFH-DA assay at 24 hpi. The fluorescence intensity was measured using a Tecan Spark microplate reader (**A**). The effects of hyperoside on MDA (**B**), SOD (**C**), and GSH (**D**) levels in these cells were also measured at 24 hpi. ^*^P < 0.05; ^**^P < 0.01; ^***^P < 0.001.

Hyperoside has previously been shown to relieve oxidative stress via the HO-1-mediated endogenous antioxidant system (12). We further explored whether the anti-EHV-8 activity of hyperoside is related to its antioxidant activity. RK-13 and NBL-6 cells were pretreated with hyperoside and then infected with EHV-8. Reactive oxygen species (ROS) levels were measured using a dichloro-dihydro-fluorescein diacetate (DCFH-DA) probe. The results showed that EHV-8 infection induces ROS elevations, whereas hyperoside treatment attenuates ROS (Fig. 7A; Fig. S1) and malondialdehyde (MDA) (Fig. 7B) levels in EHV-8-infected cells. By contrast, other biomarkers of antioxidant activity, i.e., superoxide dismutase (SOD) and glutathione (GSH), increased after hyperoside treatment (Fig. 7C and D). Notably, these elevations could be reversed by siHO-1. These data indicated that hyperoside can restore the redox balance in RK-13 and NBL-6 cells.

### Hyperoside shows anti-EHV-8 activity via HO-1-mediated IFN production

Previous studies have revealed that HO-1 is a crucial regulator of immune responses, inhibiting viral replication, and is partly associated with IFN-α/β production (22, 23).

**Fig 8:**
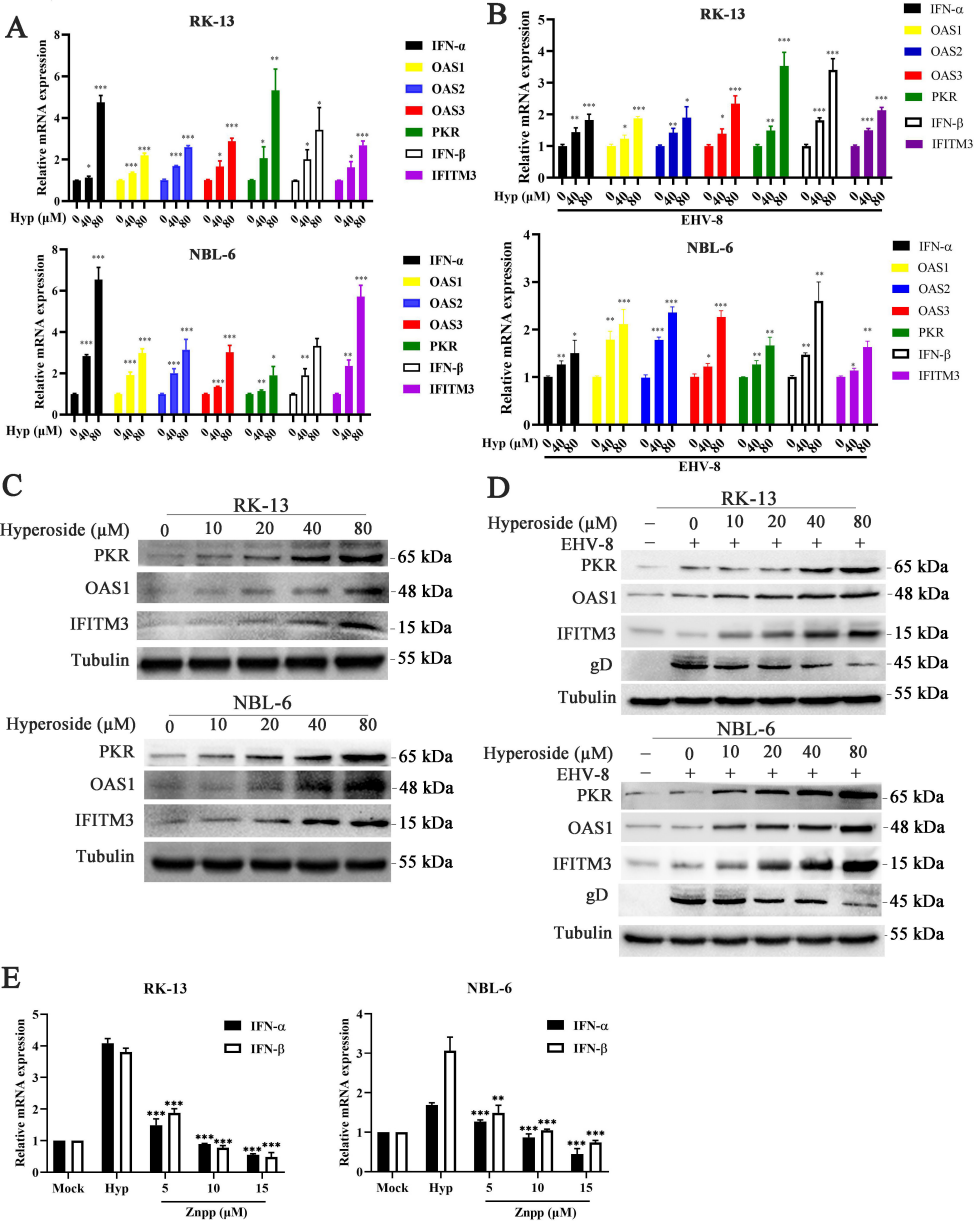
Hyperoside induced an IFN-α antiviral response. Susceptible cells were pretreated with hyperoside at the indicated concentrations for 2 h, and the mRNA expression of antiviral genes (*IFN-α*, *OAS1*, *OAS2*, *OAS3*, *PKR*, *IFN-β*, and *IFITM3*) was determined in RK-13 and NBL-6 cells without (**A**) or with (**B**) EHV-8 infection (0.1 MOI) using qPCR. GAPDH served as the internal control. ^*^P < 0.05; ^**^P < 0.01; ^***^P < 0.001, compared with 0 µM hyperoside-treated cells. Meanwhile, the protein expression of OAS1, PKR, and IFITM3 was also measured using Western blot in RK-13 and NBL-6 cells (**C and D**). (**E**) RK-13 and NBL-6 cells were incubated with a mixture of hyperoside (80 µM) and ZnPP (5, 10, and 15 µM) for 2 h, and the mRNA expression of IFN-α/β was determined using qPCR. GAPDH served as the internal control. ^*^P < 0.05; ^**^P < 0.01; ^***^P < 0.001, compared with hyperoside-treated cells.

Thus, we explored whether hyperoside could induce an IFN-α antiviral response. The expression of IFN-α-related antiviral genes in RK-13 and NBL-6 cells was examined using qPCR and Western blotting under different conditions of EHV-8 infection and hyperoside treatment. The results showed that hyperoside treatment at the indicated concentration increased the transcription of *IFN-α*, *2′−5′-oligoadenylatesynthetase1* (*OAS1*), *OAS2*, *OAS3*, *PKR*, *IFN-β*, and *IFITM3* in a concentration-dependent manner in RK-13 and NBL-6 cells, both in the absence (Fig. 8A) and presence (Fig. 8B) of EHV-8 infection. Notably, the transcription of *IFN-α*, *OAS1*, *OAS2*, *OAS3*, *PKR*, *IFN-β*, and *IFITM3* was higher in EHV-8-infected RK-13 and NBL-6 cells than in uninfected cells in the absence of hyperoside treatment (Fig. S2). Meanwhile, the protein expression of OAS1, PKR, and IFITM3 was also upregulated in RK-13 and NBL-6 cells (Fig. 8C and D).

Subsequently, RK-13 and NBL-6 cells were treated with a mixture of hyperoside and the HO-1 inhibitor ZnPP. The cells were then harvested to test IFN production using qPCR. The data showed that ZnPP reversed the upregulation of IFN-α/β induced by hyperoside in both types of cells (Fig. 8E). These findings indicated that hyperoside could trigger an IFN antiviral response against EHV-8 infection, and this effect was dependent on HO-1 activation.

### Hyperoside induces Nrf2 nuclear translocation and Keap1 degradation in NBL-6 cells

**Fig 9:**
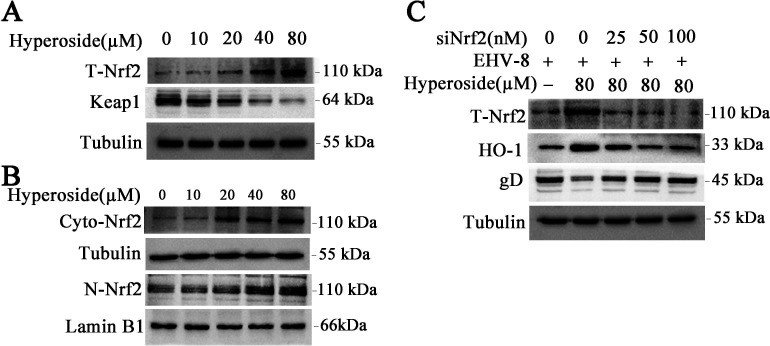
Hyperoside induced HO-1 expression via Nrf2 activation and Keap1 degradation. NBL-6 cells were pretreated with different concentrations of hyperoside or DMSO for 2 h, and the cells were then harvested to extract total cellular protein (**A**) and detect T-Nrf2 and Keap1 levels. Tubulin served as the internal control. Nuclear and cytoplasmic proteins were isolated (**B**) to detect Cyto-Nrf2, N-Nrf2, and Keap1 expression, with tubulin and Lamin B1 serving as the controls. T-Nrf2, total Nrf2 in the cell; N-Nrf2, Nrf2 in the nucleus; Cyto-Nrf2, Nrf2 in the cytoplasm. NBL-6 cells were transfected with siNrf2 at different doses, incubated with hyperoside (80 µM), and then infected with EHV-8 SDLC66 (0.1 MOI) for 24 h. These cells were collected to test Nrf2, HO-1, and gD expression using Western blot (**C**).

The Nrf2/Keap1 signaling pathway is essential for combating oxidative stress and maintaining the cellular redox balance in mammalian cells (24, 25). Nrf2 is a vital transcription regulator factor and modulates the cellular defense against oxidative stress and promotes IFN production through HO-1 upregulation (26). We first tested whether hyperoside could promote Nrf2 activation. NBL-6 cells were treated with different concentrations of hyperoside for 2 h, and the total cell lysate and nuclear fraction were harvested and analyzed using Western blot. As shown in Fig. 9A and B, hyperoside increased the accumulation of total Nrf2 and nuclear Nrf2 in NBL-6 cells in a dose-dependent manner. Meanwhile, it caused a corresponding decrease in Keap1 expression. Furthermore, NBL-6 cells were transfected with siNrf2 or siNC, treated with hyperoside (80 µM), and then infected with EHV-8. These cells were subsequently collected to evaluate Nrf2, HO-1, and gD expression. The results showed that Nrf2 expression was significantly downregulated by siNrf2. Additionally, siNrf2 induced a decrease in HO-1 levels and reversed gD expression (Fig. 9C). In summary, the results suggested that hyperoside could reduce EHV-8 replication through Nrf2-mediated HO-1 activation.

### Hyperoside activates the JNK/Nrf2/HO-1 signaling axis to inhibit EHV-8 proliferation

**Fig 10:**
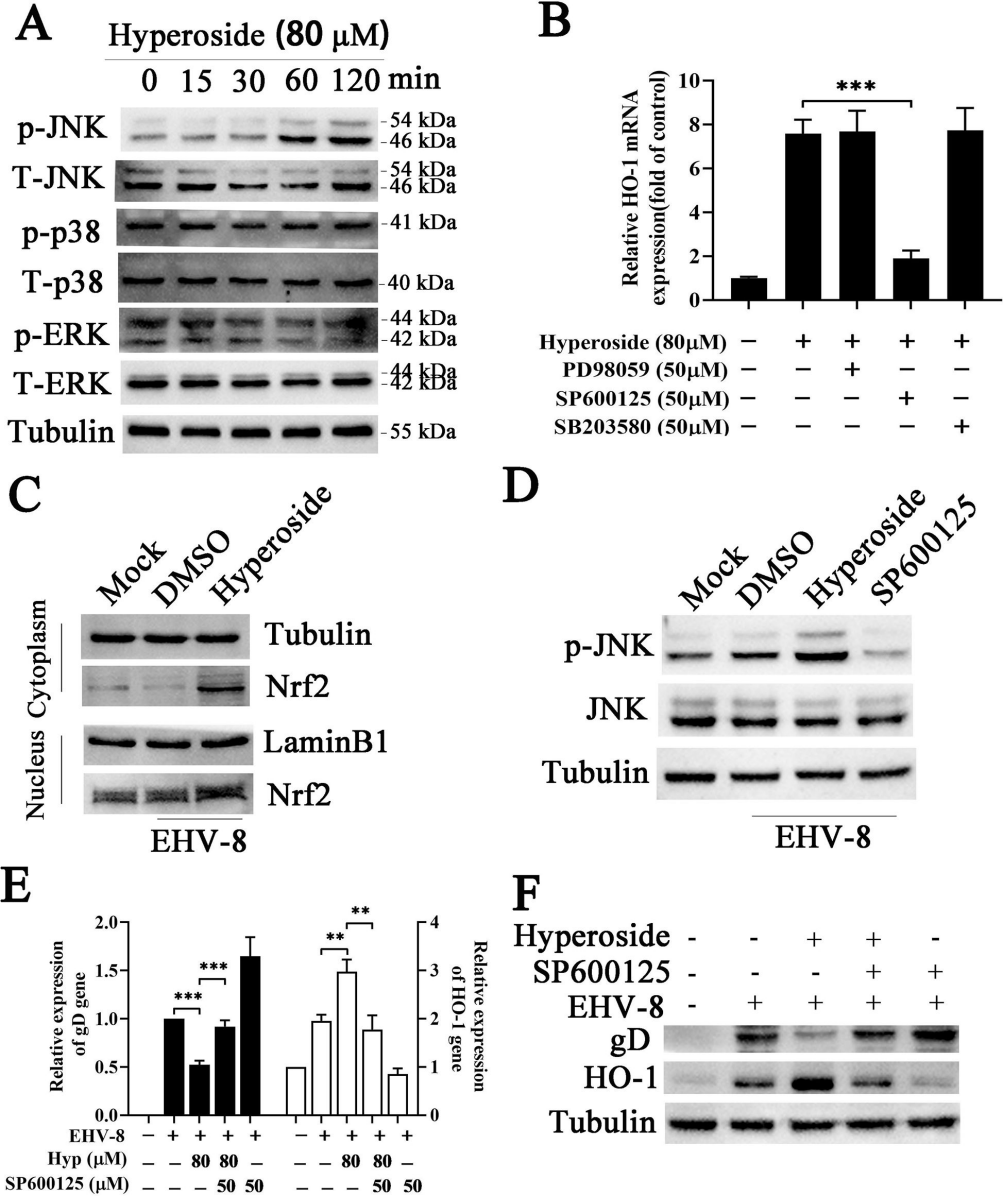
Hyperoside induced JNK-MAPK activation to stimulate the Nrf2-HO-1 pathway. (**A**) Hyperoside increased JNK phosphorylation. NBL-6 cells were incubated with 80 µM of hyperoside and harvested at the indicated time points (0, 15, 30, 60, and 120 min) to analyze the phosphorylated and total levels of the ERK1/2, JNK, and p38 proteins using Western blot. (**B**) SP600125 decreased HO-1 expression. NBL-6 cells were incubated with a JNK inhibitor (SP600125), ERK1/2 inhibitor (PD98059), and p38 inhibitor (SB203580) for 2 h, and the cells were then collected to test HO-1 expression using qPCR. ^***^P < 0.001. (**C**) Hyperoside promoted the nuclear translocation of Nrf2. NBL-6 cells were pretreated with 80 µM hyperoside for 2 h and infected with EHV-8 SDLC66 (0.1 MOI) for 1 h. The protein expression of Nrf2 in the cytoplasmic and nuclear fractions was analyzed using Western blot at 24 hpi. Tubulin and Lamin B were used as the controls. Meanwhile, the amount of p-JNK expression in the total cellular protein fraction was also determined using Western blot (**D**). NBL-6 cells were incubated with a mixture of hyperoside (80 µM) and a JNK inhibitor (SP600125) for 2 h, before infection with EHV-8 SDLC66 (0.1 MOI). The cells were collected to test HO-1 and gD expression using qPCR (**E**) and Western blot (**F**) at 24 hpi. GAPDH served as the internal control. ^**^P < 0.01; ^***^P < 0.001.

Mitogen-activated protein kinase (MAPK) signaling pathway proteins, including p38, JNK, and ERK1/2, have been found to regulate the cellular defenses against pathogen invasion (5). To investigate whether MAPKs are involved in the anti-EHV-8 effect of hyperoside, NBL-6 cells were incubated with hyperoside (80 µM) for 2 h. The phosphorylation levels of p38, ERK1/2, and JNK were analyzed using Western blot. After hyperoside treatment, a gradual time-dependent decrease in JNK phosphorylation levels was observed. Meanwhile, ERK/1/2 and p38 phosphorylation did not show significant changes at any time point (Fig. 10A). To further explore the role of JNK in the HO-1-mediated induction of hyperoside, NBL-6 cells were treated with a mixture of specific inhibitors targeting MAPKs and hyperoside, and HO-1 expression was evaluated. As shown in Fig. 10B, the JNK inhibitor SP600125 significantly decreased the HO-1 expression induced by hyperoside. In contrast, the p38 inhibitor SB203580 and ERK1/2 inhibitor PD98059 caused no significant change in HO-1 expression. In the cytoplasm and nucleus, hyperoside could induce elevations in Nrf2 levels (Fig. 10C), suggesting that it promoted the nuclear translocation of Nrf2. Furthermore, JNK phosphorylation was also increased in hyperoside-treated cells (Fig. 10D). Meanwhile, NBL-6 cells were incubated with a mixture of the JNK inhibitor SP600125 and hyperoside and subsequently infected with EHV-8. The protein expression of gD in these cells was analyzed. As expected, both gD protein and mRNA levels were higher in this group than in the group treated with hyperoside alone (Fig. 10E and F), suggesting that the JNK inhibitor SP600125 could reverse the anti-EHV-8 effect of hyperoside. These results demonstrated that the anti-EHV-8 effect of hyperoside is dependent on JNK-mediated HO-1 induction.

### Hyperoside shows anti-EHV-8 activity *in vivo*

**Fig 11:**
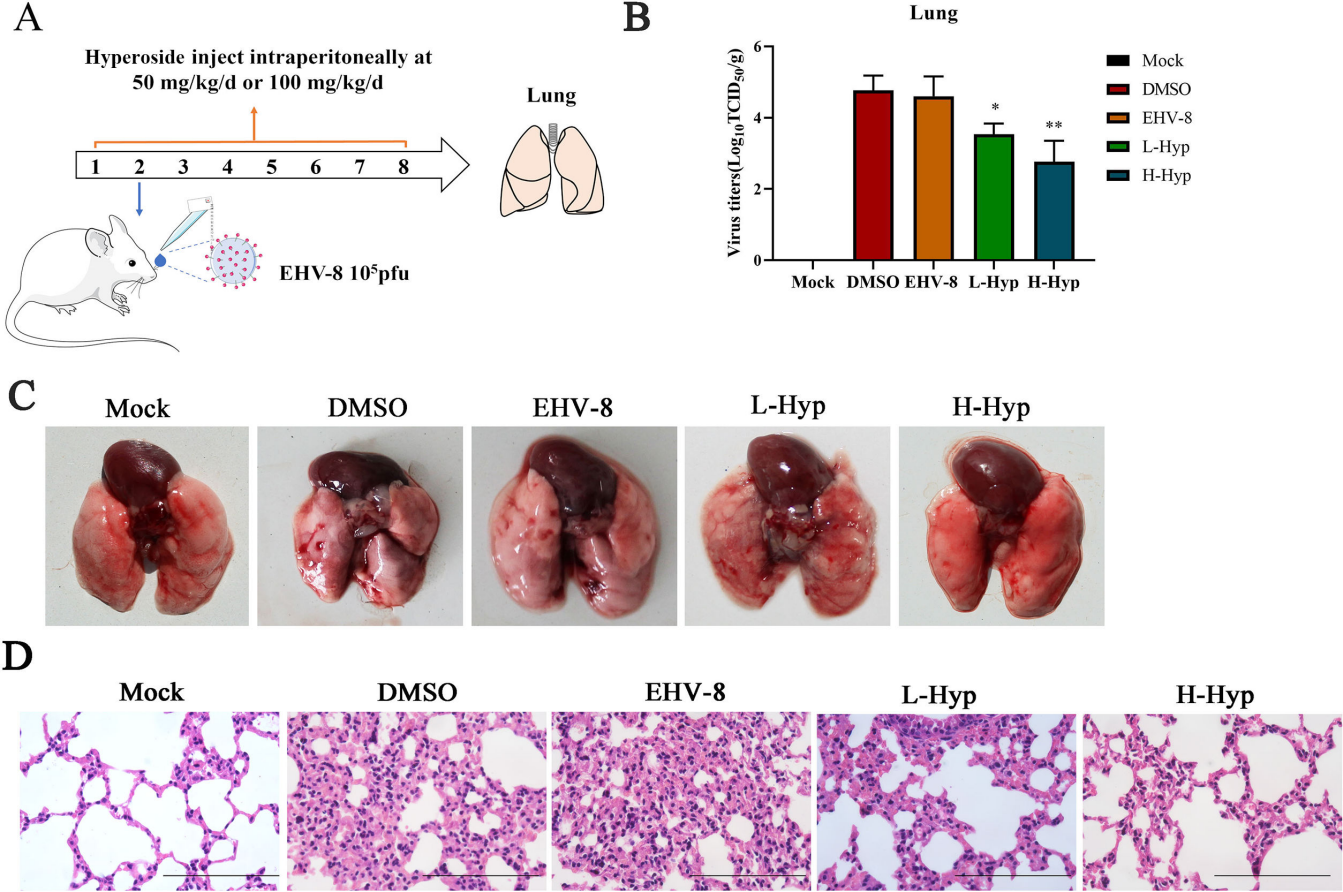
Analysis of hyperoside activity against EHV-8 infection in a BALB/c model. (**A**) Schematic diagram showing the *in vivo* evaluation of EHV-8 infection in BALB/c mice. The mice were intraperitoneally preinoculated with 50 mg/kg (L-Hyp), 100 mg/kg hyperoside (H-Hyp), or DMSO on day 1. They were then intranasally inoculated with EHV-8 (1 × 10^5^ PFU/mice) or Dulbecco's modified Eagle medium (DMEM) on day 2. Hyperoside was administered daily at the same dose after viral infection. (**B**) EHV‐8 replication in lung tissues at 7 days post-infection (dpi) was tested using a titration method in RK‐13 cells. ^*^P < 0.05; ^**^P < 0.01 compared with the DMSO-treated group. (**C**) Pathological changes in mouse lungs were observed based on necropsy findings at 7 dpi. Meanwhile, the lung tissues were fixed in 10% formalin for histopathological analysis with hematoxylin and eosin staining (**D**) (HE, ×400).

EHV-8 can cause respiratory disease and viral encephalitis in mouse models (1, 27). Our data showed that hyperoside exerts a significant antiviral effect against EHV-8 *in vitro*. Next, we explored whether hyperoside also has an antiviral effect *in vivo*. To this end, the antiviral effect of hyperoside against EHV-8 infection in BALB/c mice was investigated (Fig. 11A). The BALB/c mice were divided into five groups, as shown in Table 2. Lung tissues were collected from all mice and homogenized. The EHV-8 counts in lung tissues from different groups were examined in RK-13 cells. The mean viral titers in the hyperoside group were significantly lower than those in the DMSO group. Noticeably, H-Hyp was significantly more effective at reducing viral replication than L-Hyp (Fig. 11B). As shown in Fig. 11C, hyperoside could alleviate lung lesions after EHV-8 infection. The lungs of BALB/c mice challenged with EHV-8 SDLC66 infection for 7 days showed marked histopathological damage, characterized by large areas of alveolar wall thickening, leading to the compression and collapse of alveolar cavities and moderate inflammatory cell infiltration. In contrast, the lungs of BALB/c mice treated with hyperoside showed little to mild alveolar wall thickening and less inflammatory cell infiltration. Meanwhile, H-Hyp was more effective at attenuating the tissue damage caused by EHV-8 infection than L-Hyp (Fig. 11D). These data suggested that hyperoside can decrease EHV-8 replication in the lung tissues of infected mice, indicating its potential as a potent antiviral drug against EHV-8 infection.

### Hyperoside prevents EHV-8 infection in mice by activating HO-1 and reducing oxidative stress

**Fig 12:**
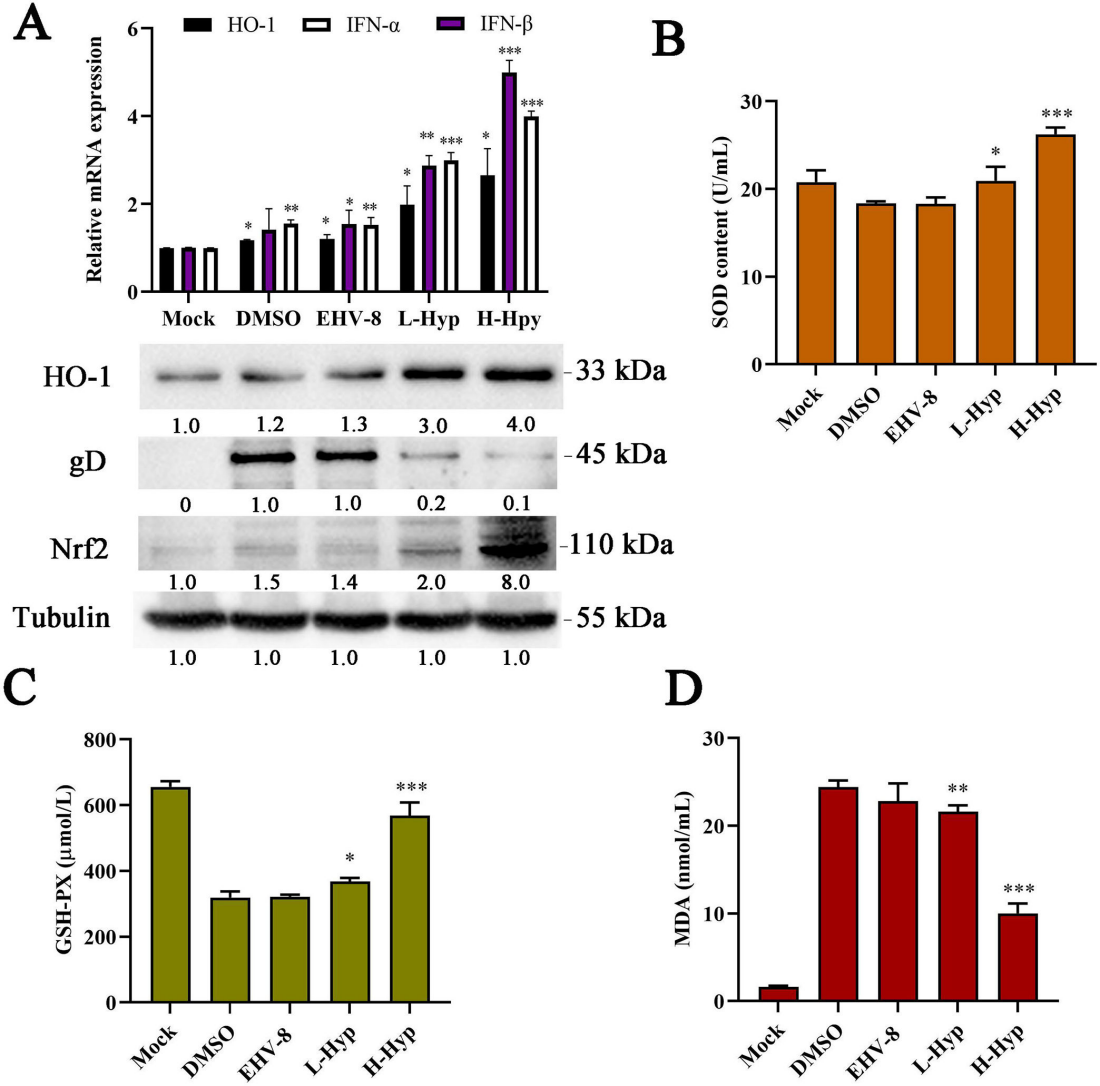
Effects of hyperoside on the expression of Nrf2, HO-1, and IFN-α/β and its impact on oxidative stress *in vivo*. RNA was extracted from the lung tissues of all groups at 7 dpi, and the transcriptional levels of IFN-α/β and HO-1 were detected using qPCR (**A**). Simultaneously, the protein expression levels of HO-1, gD, and Nrf2 were determined using Western blot (**A**). The serum levels of SOD, GSH, and MDA in each group at 7 dpi were measured using commercial kits (**B–D**). ^*^P < 0.05; ^**^P < 0.01; ^***^P < 0.001 compared with the DMSO-treated group.

To further assess the impact of hyperoside on antiviral responses and oxidative stress *in vivo*, the expression of HO-1 and IFN-α/β was determined in mouse lung tissues. As shown in Fig. 12, the transcriptional activities of IFN-α/β and HO-1 were higher in the lung tissue of the EHV-8-infected group than in the lung tissue of the mock group. Furthermore, the protein expression of HO-1 and Nrf2 was also increased (Fig. 12A). Interestingly, treatment with hyperoside significantly increased the expression of the HO-1 and Nrf2 proteins. Furthermore, it reduced gD protein expression in a dose-dependent manner when compared with DMSO treatment, while also increasing IFN-α/β transcription to enhance the antiviral response (Fig. 12A). Notably, the serum levels of SOD and GSH were lower in EHV-8-infected mice than in the mock group. Moreover, the levels of SOD and GSH were significantly higher in hyperoside-treated mice than in DMSO-treated mice. However, serum MDA levels were higher in EHV-8-infected mice than in the mock group, and hyperoside could decrease MDA levels (Fig. 12B through D). Collectively, the data indicated that hyperoside can alleviate oxidative stress and trigger IFN antiviral responses to reduce EHV-8 infection in mouse models by activating the Nrf2/HO-1 pathway.

## DISCUSSION

Previous studies have demonstrated that hyperoside has promising anti-inflammatory, antioxidant, antiapoptotic, and anticancer effects (28–30). In the present study, the potential *in vitro* antiviral activity of hyperoside was tested against different strains of EHV-8, including SDLC66, SD2020113, and donkey/Shandong/10/2021. The data revealed that hyperoside can exert anti-EHV-8 effects in RK-13, MDBK, and NBL-6 cells (Fig. 2 and 3) and can target multiple EHV-8 strains (Fig. 4).

Hyperoside is known to primarily exert anti-HBV effects by reducing HBsAg and HBeAg secretion (16). Yeh et al. reported that hyperoside isolated from honeysuckle can inhibit SARS-CoV-2 infection by decreasing viral entry and replication (31). The replication cycle of the EHV involves multiple stages, such as adsorption, viral entry, uncoating, replication, morphogenesis, and egress (32, 33). By further exploring the anti-EHV-8 mechanisms of hyperoside, we found that hyperoside could not inactivate EHV-8 directly. However, it inhibited EHV-8 infection mainly by targeting the virus at the adsorption and internalization stages (Fig. 5).

Virus-infected cells often experience damage due to oxidative stress. Recently, some drugs were found to effectively control virus infection by manipulating cellular oxidative stress responses. Sutter et al. reported that non-thermal plasma can inhibit HSV-1 replication by decreasing ROS generation (34). Moreover, Liu et al. found that xanthohumol suppresses PRRSV replication by alleviating the oxidative stress induced by PRRSV infection (35). Previously, hyperoside was reported to suppress the neuronal death caused by 6-OHDA-induced oxidative stress via Nrf2/HO-1 activation. Consistent with these findings, the present study demonstrated that hyperoside can effectively reduce EHV-8-induced oxidative stress *in vitro* (Fig. 7) and in mouse models (Fig. 12).

The innate immune system serves as the first line of defense against pathogen infection, and the type I IFN system plays an important role in controlling viral replication (36, 37). In a previous study, Oladunni et al. found that EHV-1 can strategically inhibit the host innate immune defense in equine endothelial cells by limiting type I IFN production (38). Meanwhile, Ma et al. demonstrated that CoPP, a specific activator of HO-1, decreases influenza virus replication via the IRF3-mediated production of IFN-α/β (22). A similar investigation also found that CoPP inhibits human respiratory syncytial virus replication and subsequent lung inflammation through HO-1-mediated IFN-α/β production (39). In the present study, hyperoside was found to effectively prevent EHV-8 infection by upregulating the expression of IFN-related antiviral genes (Fig. 8).

Members of the MAPK protein family serve as important kinases and modulate many cellular processes, such as inflammation, oxidative stress, antiviral responses, apoptosis, and cytoskeletal remodeling (40, 41). Nrf2, as a key transcription factor, plays an important role in inflammatory, antioxidant, and antiviral responses by regulating the expression of downstream target genes (42–44). Similarly, HO-1 is a cytoprotective protein that can modulate oxidative stress and immune responses (5, 45, 46). In recent years, the MAPK and Nrf2/HO-1 signaling pathways have been recognized as the primary regulators of intracellular defense against oxidative stress-induced injury, inflammatory responses, and viral infection (47). Celastrol has been shown to inhibit HCV replication via JNK/Nrf2/HO-1 activation (48). In line with these findings, Xing et al. reported that hyperoside can upregulate HO-1 expression to attenuate H_2_O_2_-induced cellular damage via the MAPK-dependent Keap1/Nrf2/ARE signaling pathway (49). Our findings showed that hyperoside plays an anti-EHV-8 role by activating the MAPK and Nrf2/HO-1 signaling pathways in susceptible cells. Specifically, hyperoside increases the levels of phosphorylated JNK (p-JNK), Nrf2, and HO-1 in NBL-6 cells in a time- and dose-dependent manner. Notably, these effects can be reversed by siNrf2, siHO-1, and the JNK inhibitor SP600125 (Fig. 9 and 10). Taken together, the findings indicate that hyperoside exerts antiviral effects against EHV-8 infection via JNK and Nrf2/HO-1 signaling pathway activation.

In the present study, we further verified the protective effect of hyperoside in a mouse model. Both oxidative stress responses and IFN-α/β cytokine production were examined in the lungs of mice infected with EHV-8. Our results showed that hyperoside alleviates the lung lesions induced by EHV-8 infection and decreases the viral load in the serum (Fig. 11 and 12). Thus, we speculate that hyperoside may significantly reduce EHV-8 replication *in vivo* by inducing IFN-α/β production via HO-1 activation.

**Fig 13:**
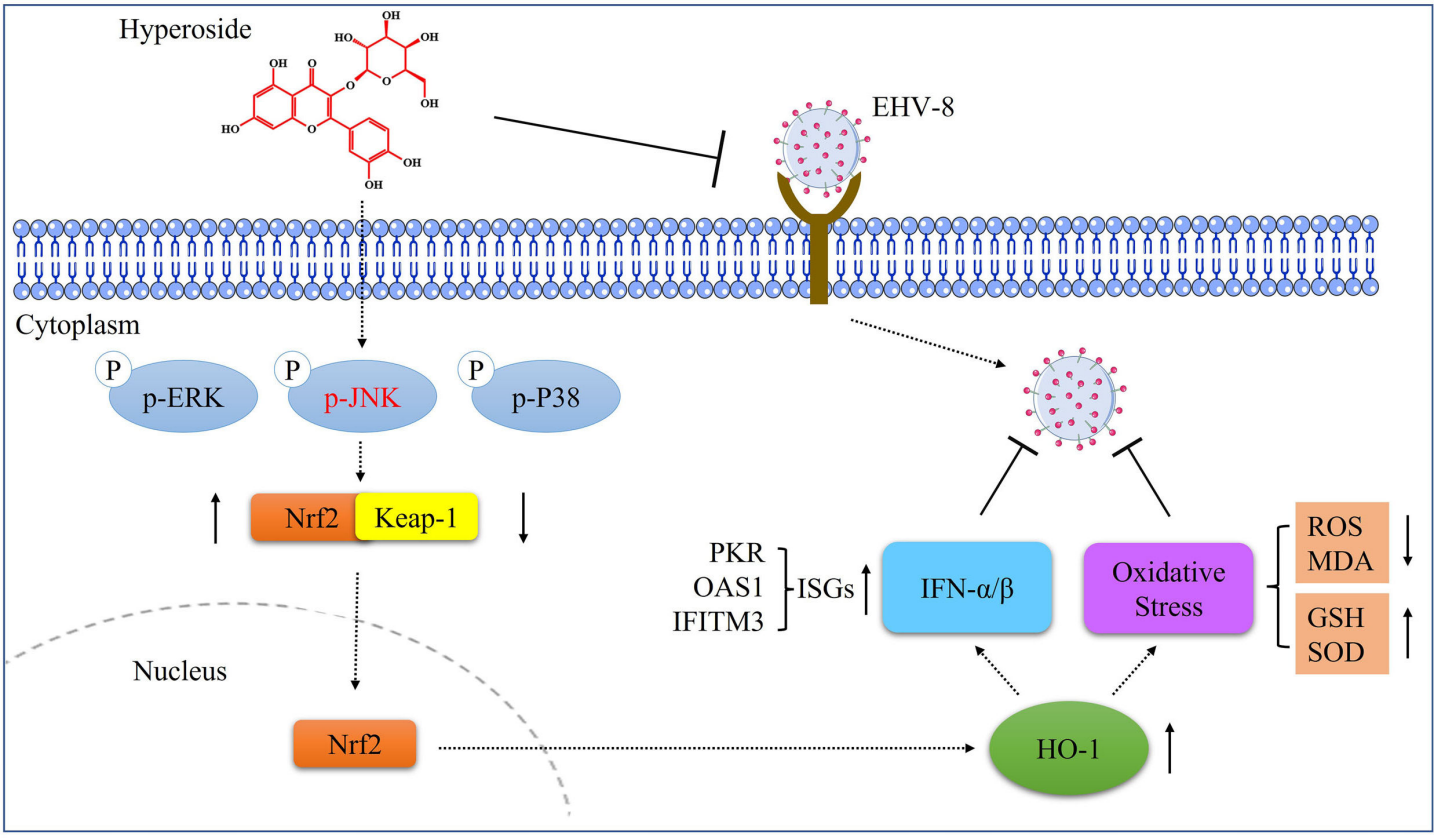
Schematic showing the mechanism of cytoprotection exerted by hyperoside against EHV-8 infection. Hyperoside activates JNK and inhibits EHV-8 adsorption and internalization into susceptible cells, upregulating Nrf2 expression and causing the subsequent activation of HO-1 gene expression. HO-1 upregulation promotes the host cellular type I IFN response, causing the induction of ISG expression. This also alleviates oxidative stress, finally resulting in the inhibition of EHV-8 replication.

Collectively, our data showed that hyperoside can inhibit EHV-8 infection *in vitro* and *in vivo*. The anti-EHV-8 mechanisms of hyperoside mainly depend on JNK/Nrf2/HO-1-mediated oxidative stress relief and IFN production (Fig. 13). The findings suggest that hyperoside could serve as a promising therapeutic agent against EHV-8 infection in the future.

## MATERIALS AND METHODS

### Cells, viruses, and reagents

RK-13 and MDBK cells were purchased from the China Center for Type Culture Collection and maintained in Modified Eagle Medium); PBS (phosphate-buffered saline (MEM) containing 10% fetal bovine serum (FBS) and penicillin–streptomycin at 37°C and 5% CO_2_. Equine dermal cells (NBL-6) were obtained from the American Type Culture Collection and cultured in MEM containing 10% FBS. The EHV-8 isolates used for this study were as follows: SDLC66 (GenBank: MW816102.1), SD2020113 (GenBank: MW822570.1), and donkey/Shandong/10/2021 (GenBank: OL856098.1). All strains were cultured in RK-13 cells, and their titers were measured using a plaque formation assay as described previously (27).

Hyperoside was purchased from Sigma Aldrich (Saint Louis, USA) and dissolved in DMSO. CoPP, a HO-1 inducer, was purchased from Frontier Specialty Chemicals (Logan, USA). Meanwhile, ZnPP, a HO-1 inhibitor, was purchased from MedChemExpress (New Jersey, USA). PD 98059 (MAPK/ERK inhibitor), SP600125 (JNK inhibitor), and SB203580 (p38 MAPK inhibitor) were purchased from Sigma (St. Louis, MO, USA). The anti-PKR antibody (catalog number 18244-1-AP) was purchased from Proteintech (Chicago, USA). The anti-OAS1 antibody (catalog number ab272492), anti-IFITM3 antibody (catalog number ab288563), anti-tubulin antibody (catalog number ab44928), and anti-Keap1 antibody (catalog number ab227828) were purchased from Abcam (Cambridge, UK). The rhodamine-conjugated goat anti-mouse IgG (catalog number 115-295-062) antibody was purchased from Jackson (Lancaster, USA). Horseradish peroxidase-conjugated anti-mouse IgG (catalog number 31430) and anti-rabbit IgG (catalog number 31460) were purchased from Thermo Fisher (Massachusetts, USA). Additionally, the anti-JNK antibody (catalog number GB114321), anti-p-p38 antibody (catalog number GB113380), anti-p38 antibody (catalog number GB114685), anti-HO-1 antibody (catalog number GB12104), anti-Lamin B1 antibody (catalog number GB111802), anti-Nrf2 antibody (catalog number GB113808), and anti-ERK1/2 antibody (catalog number GB11560) were purchased from Servicebio (Wuhan, China). Finally, the anti-p-ERK1/2 antibody (catalog number sc-81492) and anti-p-JNK antibody (catalog number sc-6254) were purchased from Santa Cruz (Dallas, USA). Mouse anti-EHV-8 serum and the mouse anti-gD polyclonal antibody were prepared in our laboratory.

### Cytotoxicity assay

The CCK-8 was obtained from Beyotime Biotechnology (Nanjing, China), and it was used to detect the cytotoxicity of hyperoside in RK-13, MDBK, and NBL-6 cells. Cells in 96-well plates were incubated with or without hyperoside for 24 h. Then, 100 µL MEM containing 10% CCK-8 reagent was added to each well, and the cells were incubated at 37°C for another 2 h. The cell viability was measured at an absorbance of 450 nm using a Spectramax Absorbance Reader (USA) and analyzed using GraphPad Prism 8.0 as previously described (50).

### Assay for testing the inhibition of viral infection

RK-13, MDBK, and NBL-6 cells were seeded on six-well plates and cultured until they reached 80%–90%. The cells were then treated with various concentrations of hyperoside (10, 20, 40, and 80 µM) or DMEM containing 0.1% DMSO for 2 h. This was followed by EHV-8 SDLC66 infection at an MOI of 0.1 for 1 h. The medium was subsequently replaced with 3% FBS MEM containing the indicated concentrations of hyperoside. The cells and cellular supernatant were collected for further Western blot and TCID_50_ analysis, respectively.

RK-13 and NBL-6 cells were seeded on 12-well plates and treated with various concentrations of ZnPP (5, 10, and 15 µM) for 10 h. We then added 80 µM hyperoside or maintenance medium containing 0.1% DMSO for 2 h, followed by infection with EHV-8 SDLC66 at a 0.1 MOI for 1 h. The medium was then replaced with 3% FBS MEM containing different concentrations of ZnPP, and the cells were collected at 24 hpi for further analysis with qPCR and Western blot.

RK-13 and NBL-6 cells were also transfected with siRNA targeting HO-1 (siRNA-HO-1-1#/2#/3#) or an siRNA negative control (siNC) for 12 h before treatment with hyperoside (80 µM) for 2 h and infection with EHV-8 SDLC66 at MOI = 0.1. These cells were harvested to examine EHV-8 replication at 24 hpi using qPCR and Western blot.

### Indirect immunofluorescence assay

The IFA was performed as previously described (51). Briefly, coverslips were pretreated in 12-well tissue culture plates. Next, RK-13, MDBK, and NBL-6 cells were seeded onto the plates and pretreated with various concentrations of hyperoside (10, 20, 40, and 80 µM) for 2 h, before infection with EHV-8 SDLC66 (MOI = 0.1). The cells were fixed with 75% cold ethanol at 36 hpi and blocked with 1% bovine serum albumin in PBS. They were then incubated with mouse anti-EHV-8 serum, which provided the primary antibodies, and later with Rhodamine-conjugated goat anti-mouse IgG as the secondary antibody. Finally, the cells were counterstained with DAPI and visualized using a fluorescence microscope (Leica DMi 8 Microsystems, Germany).

### Western blot analysis

Cells were collected, lysed with NP40 lysis buffer (Solarbio, China), and then boiled with 5× sample loading buffer. Equal amounts of protein were separated using 12% SDS-PAGE gels and transferred onto PVDF membranes, as described previously (52). The PVDF membranes were blocked with 5% non-fat dry milk, and specific antibodies were used to probe for different target proteins. Tubulin served as the control. Finally, the blots were observed and imaged using the ChemiDoc XRS imaging system (Bio-Rad, USA).

### RNA/DNA extraction and quantitative PCR analysis

Cells were collected, and their total RNA was extracted using the TRIzol reagent (Solarbio, China). Total RNA (1 µg) was reverse-transcribed into cDNA using the PrimeScript RT Master Mix (Takara, Japan), and qPCR was performed using the 2× RealStar Green Fast Mixture (GenStar, China). GAPDH served as the internal reference gene, and its transcripts were amplified to normalize the total RNA input. The relative quantification of target genes was performed using the 2^−∆∆Ct^ method, as described previously (51). Sequences of the primers used for qPCR are listed in Table 1.

**TABLE 1:** The primer sequences used for qPCR

Gene name	Forward primer sequences (5′−3′)	Reverse primer sequences (5′−3′)
GAPDH	CCTTCCGTGTCCCTACTGCCAAC	GACGCCTGCTTCACCACCTTCT
ORF-72	CCCACGTGTGCAACGCCTAT	ATACAGTCCCGAGGCAGAGT
HO-1	AGTTCATGAAGAACTTTCA	TACCAGAAGGCCATGTCC
IFN-α	TACTCAGCAGACCTTGAACCT	CAGTATTGGCAGCAAGTTGAC
OAS1	GGAGGCGGTTGGCTGAAGAGG	GAACCACCGTCGGCACATCC
OAS2	CCGGGCCAGTGCACAAGTTAG	CGATGGCACCGAGGACACC
OAS3	TCTGGGGTCGCTAAACATCAC	GATGACGAGTTCGACATCGGT
PKR	CGTTTCTTGCCTCCTGCTTTG	GGGACCTCCACATGACAGAAG
IFN-β	AGCTCCAAGAAAGGACGA	GCCCTGTAGGTGAGGTTGATCT
β-Actin	ACGGCATCGTCACCAACTG	CAAACATGATCTGGGTCATCTTCTC
siRNA-HO-1–1#	GGTCCTCACACTCAGCTTT	
siRNA-HO-1–2#	CCACCAAGTTCAAGCAGCT	
siRNA-HO-1–3#	AGGACATGGCCTTCTGGTA	
siRNA-Nrf2-1#	CTCCTTAAGAAGCAACTCA	
siRNA-Nrf2-2#	GTCACTCTCTGAACTTCTA	
siRNA-Nrf2-3#	GACATTCCCATTTGTAGAT	

To detect the viral copies of EHV-8 in the cell supernatant, a fragment of gD (*ORF72* gene) was amplified using the *ORF72*-F and *ORF72*-R primers and cloned into the pMD18-T vector to generate the recombinant plasmid pMD18-T-gD. This plasmid was used to develop a standard curve. Furthermore, the PCR assay for absolute quantification was performed as described previously (27, 53). Briefly, DNA was extracted from the virus particles in the supernatant using the DNA Viral Genome Extraction Kit (Solarbio, China), and the DNA copies of EHV-8 were detected using qPCR and enumerated via normalization with the standard curve.

### Viral titration

The production of viral progeny was measured in RK‐13 cells using the Reed–Muench method as described previously (1). Briefly, the cells were grown to approximately 80%–90% confluence in 96-well cell plates (Corning, USA). The viral supernatants were serially diluted 10-fold, and 100 µL of each dilution was added to each well. After 1 h of virus adsorption, the cells were washed with PBS and incubated in 3% FBS DMEM. Five days post-infection, the TCID_50_ was calculated and analyzed using GraphPad 8.0.

### Time course analysis of hyperoside’s activity against EHV-8

To determine which stage of the EHV-8 life cycle is affected by hyperoside, RK-13 and NBL-6 cells were seeded into 12-well plates. The cells were treated with hyperoside (80 µM) before, during, or after EHV-8 SDLC66 (0.1 MOI) inoculation (pretreatment, co-treatment, and post-treatment, respectively). After 24 h, qPCR and Western blot assays were performed to examine EHV-8 replication in these cells as described above.

For the direct inactivation assay, RK-13 and NBL-6 cells were seeded into 12-well plates after the cell confluence reached 80%. EHV-8 SDLC66 (0.1, 0.5, and 1 MOI) was incubated with hyperoside (80 µM) at 37°C for 1  h. Then, the pretreated virus was incubated with the cells for 1 h. Finally, the cells and the cellular supernatant were collected at 24 hpi to measure EHV-8 replication using qPCR.

For the virus adsorption assay, RK-13 and NBL-6 cells were cultured in 12-well plates and prechilled at 4°C for 1 h. A mixture of hyperoside (80 µM) or DMSO and EHV-8 (1 MOI) was added to the cells, and the cells were incubated at 4°C for another hour. The cells were washed with precooled PBS to remove unadsorbed virus particles and kept at 37°C for 24 h. Viral copies of EHV-8 were finally detected using qPCR.

For the virus internalization assay, RK-13 and NBL-6 cells were seeded in 12-well plates and grown to approximately 80% confluence. They were pretreated with hyperoside (80 µM) for 12 h, washed, and then incubated with EHV-8 (0.1 MOI) for 1 h at 4°C to allow virus attachment. The cells were subsequently washed with ice-cold PBS to remove unbound virus particles. Thereafter, the culture medium was replaced with DMEM containing 80 µM hyperoside or DMSO, and the cells were incubated at 37°C for 1 h. Cells were washed with citrate buffer (pH 3.0) to remove non-internalized virus particles and incubated at 37°C. Viral copies of EHV-8 were detected at 24 hpi using qPCR.

### Assays for ROS, MDA, GSH, and SOD activity

The cellular levels of ROS in EHV-8-infected cells were tested using a dichlorofluorescein ROS assay kit (Beyotime Biotechnology, China). Briefly, RK-13 and NBL-6 cells were transfected with siHO-1 for 10 h and then pretreated with 80 µM hyperoside or DMSO for 2 h. The cells were infected with EHV-8 (0.1 MOI) for another 1 h. Subsequently, at 24 hpi, 10 µM DCFH-DA or 50 µg/mL Rosup (positive control) was added to the cells. The cells were incubated in this reagent for 25 min at 37°C. After washing with PBS, the fluorescent intensity of the cells was measured using a Tecan Spark microplate reader (Austria). Finally, the data were analyzed using GraphPad Prism. Images were acquired on a Leica DMi8 fluorescence microscope using Leica X software.

The levels of GSH, SOD, and MDA in EHV-8-infected cells and mouse serum were determined using a glutathione peroxidase assay kit, a SOD assay kit, and a microscale MDA assay kit, respectively, according to the manufacturer’s instructions (Jiancheng Bioengineering Institute, China). The levels were normalized to the protein concentration determined using a Pierce BCA protein assay kit (Thermo, USA). The values were calculated using BioTek Epoch (BioTek, USA).

### Anti-EHV-8 assay *in vivo*

Twenty-five male, specific pathogen-free BALB/c mice (8 weeks old) were purchased from Pengyue Laboratory Animal Breeding Co., Ltd (Jinan, China) and randomly divided into five groups (*n* = 5 mice/group), as shown in Table 2. Mice were intraperitoneally preinoculated with the indicated dose of hyperoside or DMSO. They were then intranasally inoculated with EHV-8 (1 × 10^5^ PFU/mice) or DMEM. After viral infection, hyperoside was administered daily at the same dose. Each group of mice was housed separately to prevent cross-infection. The mice were observed daily for any clinical signs of infection, and their body weight was also monitored. Finally, at 7 dpi, the mice were euthanized via cervical dislocation. A careful postmortem examination was immediately performed to collect lung tissue and serum samples for further analysis.

**TABLE 2:** Group design for hyperoside treatment in mouse model

Groups	Inoculation
Mock	100 µL DMEM
DMSO	DMSO + 100 µL EHV-8
EHV-8	100 µL EHV-8
High hyperoside (H-Hyp)	Hyperoside (100 mg/kg) + 100 µL EHV-8
Low hyperoside (L-Hyp)	Hyperoside (50 mg/kg) + 100 µL EHV-8

### Pathological examination

Histopathological assays were performed as described previously (1). Briefly, the lung tissues from different groups of mice were fixed in 10% formalin, dehydrated, embedded in paraffin, and sectioned to 4 µm thickness. The sections were stained with hematoxylin and eosin and observed under a light microscope.

### Statistical analysis

The data were analyzed using GraphPad Prism 8.0 (GraphPad software, San Diego, CA, USA). All data were presented as the mean ± standard deviation of at least three independent experiments. Differences among the groups were analyzed using one-way ANOVA followed by Bonferroni’s *post hoc* test or unpaired Student’s *t*-tests. *P*-values <0.05 and *P-*values <0.01 were considered to indicate statistically significant and very statistically significant results.

## Data Availability

The original data obtained in the study are included in the article. Further inquiries can be directed to the corresponding authors.

## SUPPLEMENTAL MATERIAL

Fig. S1 jvi.00159-24-s0001.tif ROS generation in RK-13 and NBL-6 cells.

Fig. S2 jvi.00159-24-s0002.tif IFN-relative gene expression induced by EHV-8 infection.

Supplemental legends jvi.00159-24-s0003.docx Legends for Fig. S1 and S2.

ASM does not own the copyrights to Supplemental Material that may be linked to, or accessed through, an article. The authors have granted ASM a non-exclusive, world-wide license to publish the Supplemental Material files. Please contact the corresponding author directly for reuse.
