# Supplementary figures and images for: Hyperoside inhibits EHV-8 infection via alleviating oxidative stress and IFN production through activating JNK/Keap1/Nrf2/HO-1 signaling pathways
Source: J Virol. 2024 Mar 19;98(4):e00159-24. doi: 10.1128/jvi.00159-24 (PMC11019850; doi:10.1128/jvi.00159-24)

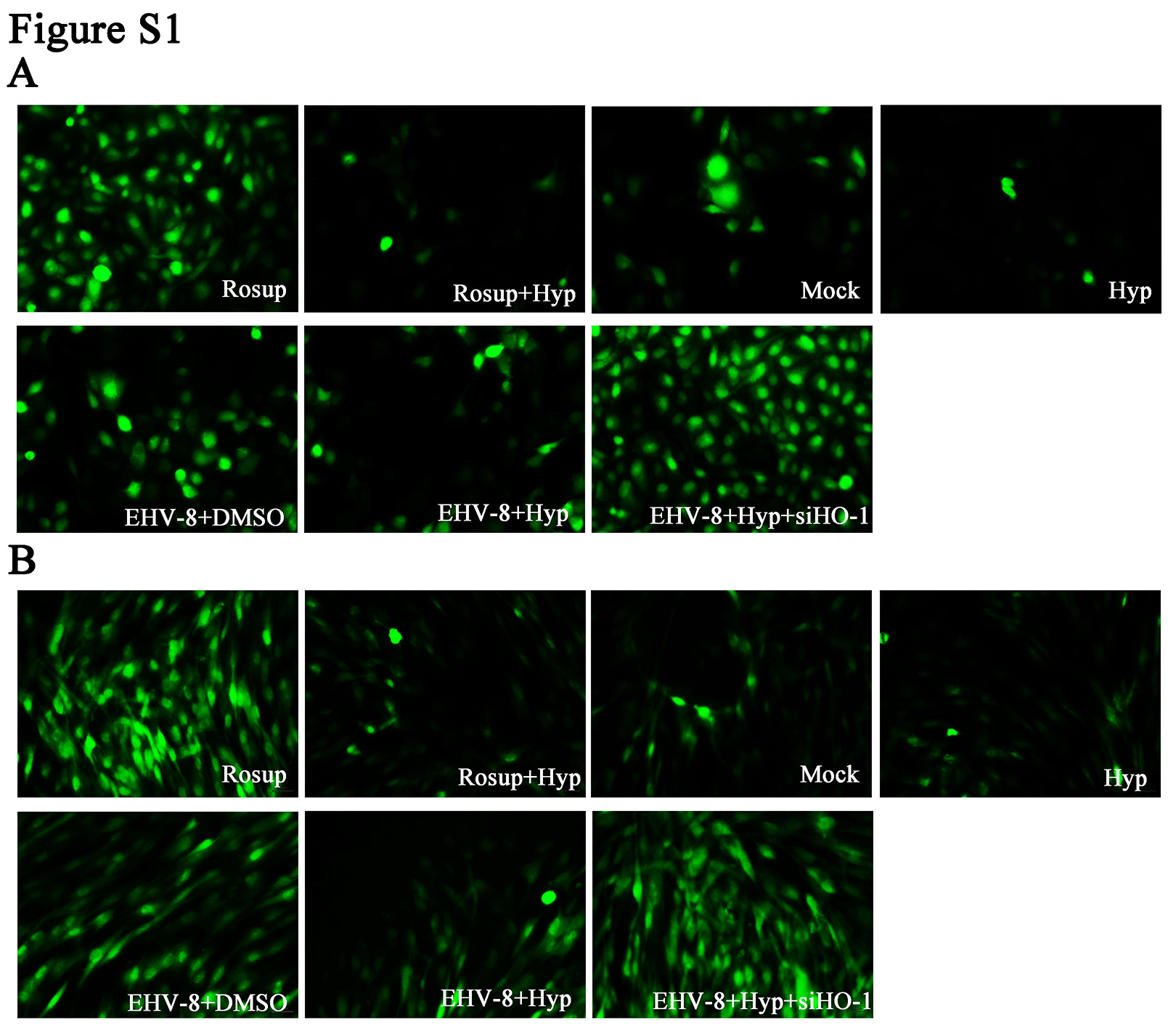

Supplement: Fig. S1 — ROS generation in RK-13 and NBL-6 cells. [file jvi.00159-24-s0001.tif]

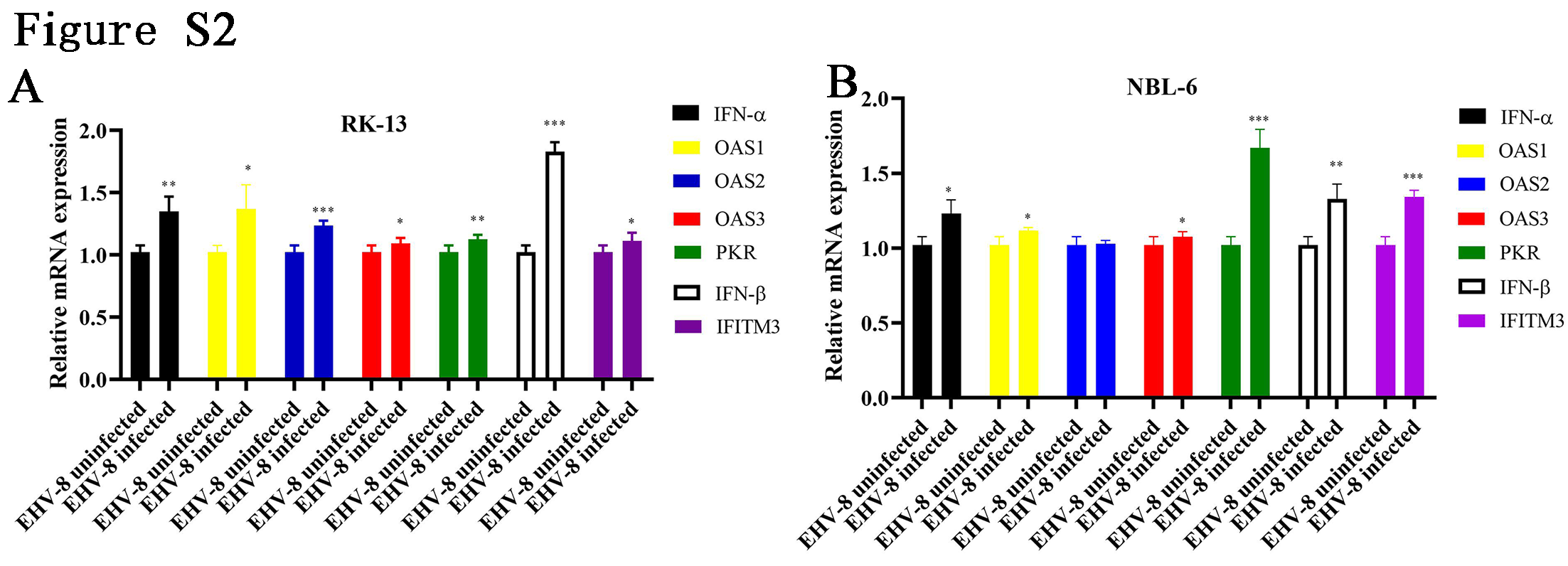

Supplement: Fig. S2 — IFN-relative gene expression induced by EHV-8 infection. [file jvi.00159-24-s0002.tif]
