# Supplementary material for: Hyperoside inhibits EHV-8 infection via alleviating oxidative stress and IFN production through activating JNK/Keap1/Nrf2/HO-1 signaling pathways
Source: J Virol. 2024 Mar 19;98(4):e00159-24. doi: 10.1128/jvi.00159-24 (PMC11019850; doi:10.1128/jvi.00159-24)
Supplement: Supplemental legends — Legends for Fig. S1 and S2. [file jvi.00159-24-s0003.docx]

Supplemental Figure legends

**Figure S1 ROS generation in RK-13 and NBL-6 cells.** RK-13 (A) and NBL-6 (B) cells were treated with indicated drug or EHV-8, ROS generation were detected using dichlorofluorescein (DCF), images were acquired by using Leica DMi8 fluorescence microscope, and the images were recorded using Leica X software.

Figure S2 IFN-relative gene expression induced by EHV-8 infection. RK-13 **(A)** and NBL-6 **(B)** were infected with or without EHV-8 (0.1 MOI), the expression of antiviral genes (IFNα, OAS1, OAS2, OAS3, PKR, IFNβ, and IFITM3) in mRNA level was determined by qPCR at 24 hpi. GAPDH served as an internal control. *, *P* < 0.05; **, *P* < 0.01; ***, *P* < 0.001, compared with EHV-8 uninfected cells.
